# Supplementary material for: Effect of hemodialysis on short-term outcomes after colon cancer surgery
Source: PLoS One. 2022 Jan 12;17(1):e0262531. doi: 10.1371/journal.pone.0262531 (PMC8754322; doi:10.1371/journal.pone.0262531)
Supplement: Attachment — Submitted filename: renamed_b4696.docx [file pone.0262531.s003.docx]

**Response to reviewers’ comments**

November 30, 2021

Robert Jeenchen Chen, MD, MPH

Academic Editor

*PLOS ONE*

Dear Dr. Chen,

Thank you for considering our paper entitled “Effect of hemodialysis on short-term outcomes after colon cancer surgery” (PONE-D-21-10207) for publication in *PLOS ONE*, subject to revision. We have revised our manuscript according to the helpful comments provided by the reviewers, addressed the concerns raised, and accepted their valuable advice.

Changes in the revised manuscript are shown in bold red font. Our point-by-point responses to the reviewers’ comments are provided below.

**Response to Reviewer 3**

**Major**

#1. Why did the authors include those complications? What are they based on?

**Response:**

We chose the postoperative complications listed in the Clavien Dindo (CD) classification. This time, we focused on CD grades 2 and higher because these are clinically problematic. We have added the following sentence to the Materials and methods section: “This time, we focused on CD grades 2 and higher because these are clinically problematic.”

#2. The authors mentions that the variables with a p value < 0.2 in the univariate analysis were included in the multivariate analysis. That is, in the regression analysis in table 4, they only included HD and operation time in the multivariate model. As shown in their table 2, HD group is associated with comorbidities, which could be confounders and should be adjusted in the regression model. In addition, anemia and malnutrition were also risk factors of post-operational complications. I suggest the authors also to collect lab data such as Hb and albumin and adjust these potential confounders.

**Response:**

Thank you for this important comment. We calculated variance inflation factors (VIFs) to detect multi-collinearity among the predictors in our regression models. The VIFs were 1.902 (sex), 2.355 (age), 1.279 (BMI), 3.214 (ASA-PS), 1.039 (HD), 1.678 (tumor location), 1.168 (preoperative chemotherapy), 1.832 (clinical T), 2.131 (clinical N), 1.375 (clinical M), 1.345 (combined resection), 1.272 (surgical approach), and 2.132 (operation time). Therefore, we consider that multi-collinearity is not a significant problem in our model. We agree with the reviewer that Hb and albumin are very important factors. However, it is difficult to collect additional blood test data because we have dealt with data from multiple facilities this time. Therefore, we will mention this point as a limitation.

In response, we have added the following to the limitation section: “Although Hb and albumin are also very important factors, it is difficult to collect additional blood test data because the present data were collected from multiple facilities. However, we calculated variance inflation factors (VIFs) to detect multi-collinearity among the predictors in our regression model. The VIFs were 1.902 (sex), 2.355 (age), 1.279 (BMI), 3.214 (ASA-PS), 1.039 (HD), 1.678 (tumor location), 1.168 (preoperative chemotherapy), 1.832 (clinical T), 2.131 (clinical N), 1.375 (clinical M), 1.345 (combined resection), 1.272 (surgical approach), and 2.132 (operation time). Therefore, we consider that multi-collinearity is not a significant problem in our model.”

**Minor**

#1. The authors should mention what comorbidity they included in the method session or table 1 and table 2.

**Response:**

Table 1 lists all comorbidities that were assessed, including hypertension, diabetes, dyslipidemia, heart disease, and cerebrovascular disease. Table 2 shows the presence or absence of the same comorbidities as in Table 1, except for HD and renal dysfunction. We have clarified this point in the Materials and methods section.

#2. In table 4, the authors divided continuous variables such as age, BMI, and operation time into binary variables. They also divided the ordinal variable ASA performance status into binary variable. What do they based on to decide the cut points?

**Response:**

In Japan, ‘elderly’ is defined as age 65 and over, but many people have good PS even if they are aging and have comorbidities. Therefore, it is common in Japan that those aged 70 and over are considered ‘elderly’ when comparing the results statistically.

The BMI considered to indicate obesity differs among countries. In Japan, BMI ≥25 is generally used as the standard, and for this reason we set the BMI cutoff point to 25.

Regarding operation time, we decided the cutoff point using the ROC curve tool of Bell Curve for Excel software.

We divided ASA-PS into 1 and 2 or more to enable comparison of patients who had good general condition without comorbidities with those who had some comorbidities.

#3. In the methods session, the author indicated the definitions of CKD. What did they mean by “obvious renal dysfunction in pathological examination”? How are the estimated glomerular filtration rates calculated?

**Response:**

Thank you for this important comment. Many kidney diseases are confirmed by histopathology, and the histopathological findings lead to estimation of renal function prognosis and treatment policy decisions. I apologize that this sentence was incorrect. We have replaced “obvious renal dysfunction” with “obvious kidney disease”. In Japan, the following formula is generally used to calculate eGFR:

eGFR = **194 × serum Cr^-1.094^ × age^-0.287^ (× 0.739 for females).**

**Please also note that we have identified an error in Table 1. Although not directly related to the main focus of this article, the number has been corrected. We apologize for this oversight.**

I confirm that all authors have approved the revised manuscript. We hope that we have satisfied the concerns of the reviewers, and that the manuscript will now be considered suitable for publication in *PLOS ONE*. Please feel free to contact me if any questions arise.

Sincerely,

Tetsuro Tominaga, MD, PhD

Department of Surgical Oncology

Nagasaki University Graduate School of Biological Sciences

1-7-1 Sakamoto, Nagasaki 852-8501, Japan

Phone: +81-95-819-7304 Fax: +81-95-819-7306

E-mail: tetsuro.tominaga@nagasaki-u.ac.jp
